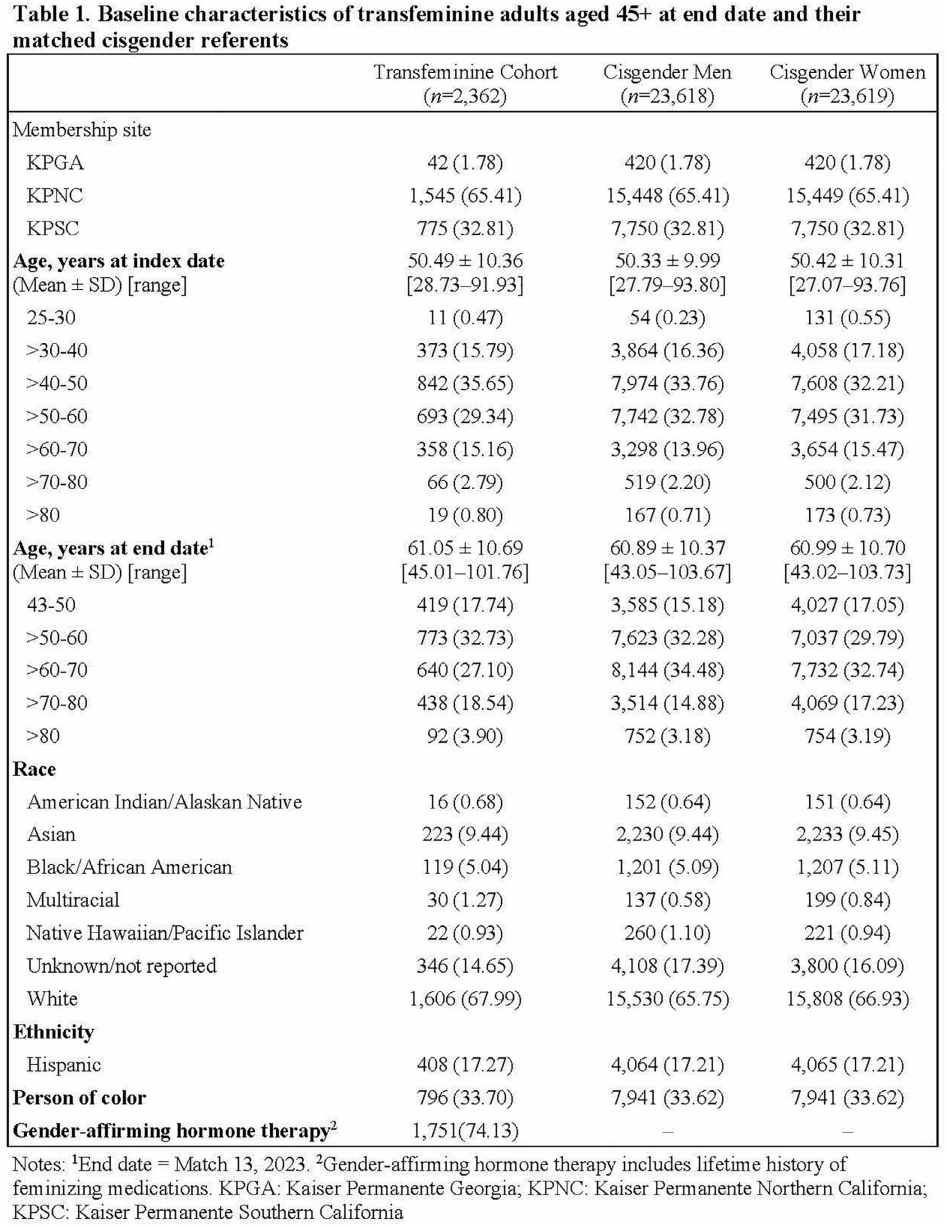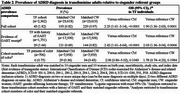# Alzheimer's disease and related dementias among transfeminine adults: A cohort study

**DOI:** 10.1002/alz70860_098087

**Published:** 2025-12-23

**Authors:** Ethan C. Cicero, Jason D. Flatt, Vin Tangpricha, Darios Getahun, Courtney McCracken, Michael J. Silverberg, Suma Vupputuri, Michael Goodman

**Affiliations:** ^1^ Emory University, Atlanta, GA, USA; ^2^ University of Nevada Las Vegas School of Public Health, Las Vegas, NV, USA; ^3^ Kaiser Permanente Southern California, Pasadena, CA, USA; ^4^ Kaiser Permanente Bernard J. Tyson School of Medicine, Pasadena, CA, USA; ^5^ Kaiser Permanente Georgia, Atlanta, GA, USA; ^6^ Kaiser Permanente, Northern California, Oakland, CA, USA; ^7^ Kaiser Permanente Mid‐Atlanta States, Rockville, MD, USA

## Abstract

**Background:**

Our understanding of Alzheimer's disease and related dementias (ADRD) among transgender people is limited. For example, it is unclear if ADRD is more common among transfeminine (TF) adults or among TF adults using gender‐affirming hormone therapy (GAHT) relative to cisgender adults.

**Methods:**

Electronic health record data (January 2006–March 2023) were used to identify a cohort of TF individuals (*n* = 2,362) aged 45+ enrolled in integrated health systems in California and Georgia. Each transgender individual was matched to 10 cisgender men (CM) and 10 women (CW) on birth year, race/ethnicity, study site, and enrollment at index date (first evidence of transgender status in TF cohort members). Odds ratios (OR) along with 95% confidence intervals (CI) were calculated to compare ADRD prevalence among TF adults compared to CM and CW referents. The same analyses were performed using two subsets of the TF cohort: persons with evidence of GAHT receipt and persons of color. Each subset was compared to their respective reference groups.

**Results:**

TF adults had a higher prevalence of ADRD (1.8%) relative to CM (0.8%) and CW (0.9%). The odds of ADRD among TF were about double the corresponding odds in CM (OR=2.3; 95% CI: 1.6–3.1) and CW (OR=1.9; 95% CI: 1.4–2.6). When restricting analyses to TF with evidence of GAHT and their cisgender referents, the associations were only slightly stronger than in the overall analysis with OR (95% CI) estimates of 2.4 (1.6–3.6) and 2.0 (1.4–3.0) related to CM and CW, respectively. The results restricted to cohort members of color were similar, but imprecise due to small numbers of ADRD cases in this group.

**Conclusion:**

Whereas TF adults have significantly higher prevalence of ADRD compared to demographically similar cisgender referents, this association does not appear to be attributable to GAHT use. Future ADRD research should take a life‐course perspective and examine the role of known modifiable risk factors such as social isolation, high cholesterol, head injury, physical inactivity, smoking, excessive alcohol consumption, hypertension, obesity, and depression in this population.